# Intestinal Barrier Function and Immune Homeostasis Are Missing Links in Obesity and Type 2 Diabetes Development

**DOI:** 10.3389/fendo.2021.833544

**Published:** 2022-01-25

**Authors:** Sylvia Riedel, Carmen Pheiffer, Rabia Johnson, Johan Louw, Christo J. F. Muller

**Affiliations:** ^1^ Biomedical Research and Innovation Platform, South African Medical Research Council, Tygerberg, South Africa; ^2^ Centre for Cardio-Metabolic Research in Africa, Division of Medical Physiology, Department of Biomedical Sciences, Faculty of Medicine and Health Sciences, University of Stellenbosch, Tygerberg, South Africa; ^3^ Department of Obstetrics and Gynaecology, University of Pretoria, Pretoria, South Africa; ^4^ Department of Biochemistry and Microbiology, University of Zululand, KwaDlangezwa, South Africa

**Keywords:** intestinal immune system, intestinal barrier function, type 2 diabetes, obesity, leaky gut, intestinal epithelial cells

## Abstract

Noncommunicable diseases, such as type 2 diabetes (T2D), place a burden on healthcare systems worldwide. The rising prevalence of obesity, a major risk factor for T2D, is mainly attributed to the adoption of Westernized diets and lifestyle, which cause metabolic dysfunction and insulin resistance. Moreover, diet may also induce changes in the microbiota composition, thereby affecting intestinal immunity. The critical role of intestinal immunity and intestinal barrier function in the development of T2D is increasingly acknowledged, however, limited studies have investigated the link between intestinal function and metabolic disease. In this review, studies reporting specific roles of the intestinal immune system and intestinal epithelial cells (IECs) in metabolic disease are highlighted. Innate chemokine signaling, eosinophils, immunoglobulin A (IgA), T helper (Th) 17 cells and their cytokines were associated with obesity and/or dysregulated glucose homeostasis. Intestinal epithelial cells (IECs) emerged as critical modulators of obesity and glucose homeostasis through their effect on lipopolysaccharide (LPS) signaling and decontamination. Furthermore, IECs create a link between microbial metabolites and whole-body metabolic function. Future in depth studies of the intestinal immune system and IECs may provide new opportunities and targets to develop treatments and prevention strategies for obesity and T2D.

## 1 Introduction

Non-communicable diseases (NCDs) such as type 2 diabetes (T2D), are a leading cause of morbidity and mortality, with increasing incidence in the developing world (1). In 2019, approximately 463 million adults were living with diabetes worldwide, and this number is said to increase to 700 million by 2045 (2). Sub-Saharan Africa has the highest proportion of undiagnosed diabetes cases in the world (~60%) and it is estimated that 73% of deaths due to diabetes occur in individuals younger than 60 years. Projections indicate that diabetes prevalence in Africa will increase by 143% over the next 24 years (2), the most significant increase globally.

The rising T2D prevalence in Africa is mainly attributed to obesity and rapid urbanization, which is associated with the adoption of unhealthy lifestyles, characterized by energy-dense diets and physical inactivity. The fundamental role that diet plays in human health and well-being is appropriately expressed by the phrase “We are what we eat”, derived from the original quote in French “Dis-moi ce que tu manges et je te dirai qui tu es” (“Tell me what you eat: I tell you who you are”) that has been traced back to Jean Anthelme Brillat-Savarin and a book, which was first published in 1826 (3, 4). An unhealthy diet is arguably the greatest modifiable risk factor for obesity and T2D (5, 6). The westernized diet, which is a high caloric diet characterized by a high fat and high sugar content (7), leads to chronic subclinical inflammation, which, in turn, has been associated with obesity and T2D (8–11). Several lines of evidence demonstrate that overnutrition leads to adipocyte dysfunction and inflammation, which are considered primary mechanisms linking diet to metabolic disease (6, 12, 13).

**Figure 1:**
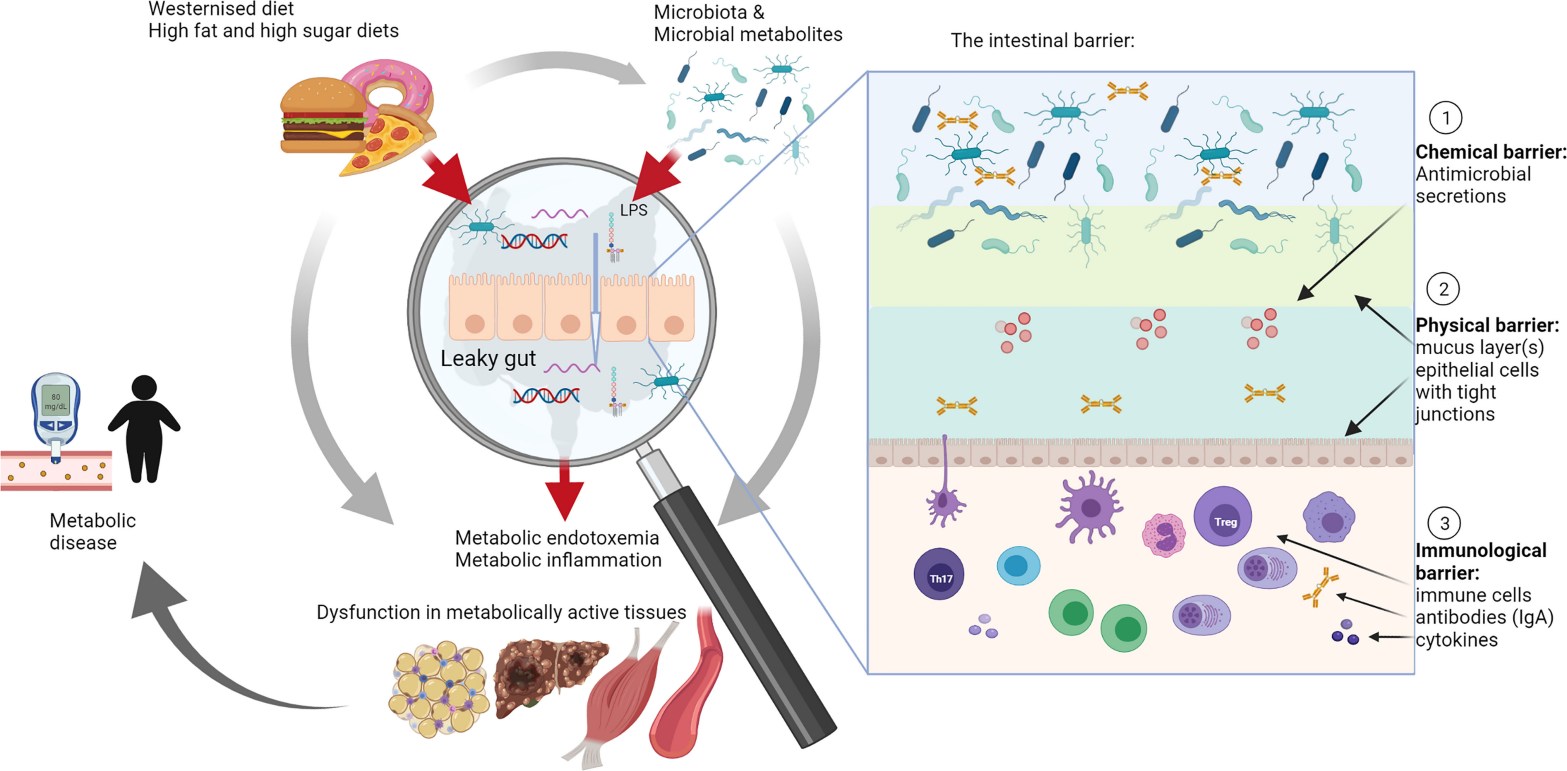
The role of intestinal barrier dysfunction and leaky gut syndrome in metabolic disease. A high fat and/or westernized diet affects and/or changes microbiota composition. Microbiota composition was associated with metabolic diseases in a large number of studies that investigated these in conjunction with metabolic outcomes, such as increased or decreased body weight, changes in glucose metabolism and effects on metabolically active tissues such as adipose tissue, liver and muscles (grey arrows) (18, 23–28). However, increasingly research is focusing on the role of the leaky gut, particularly investigating the role of the intestinal barrier and its different layers, including chemical, physical and immunological barriers (see insert), as mediators of interactions with diet and microbiota in the development of metabolic diseases (red arrows). Studies in this area are relatively limited, but this review summarizes compelling evidence for a leading role of the gut in development of metabolic disease through modulation of this complex intestinal barrier. IgA, immunoglobulin A; LPS, lipopolysaccharide; Th17, T helper cells 17; Treg, regulatory T cells.

In recent years, the role of the intestine in the development of obesity and T2D is increasingly recognized. Studies conducted in various animal models provide convincing evidence that inflammation originates in the intestine due to the modulation of gut barrier function leading to metabolic endotoxemia (14–18). Metabolic endotoxemia refers to a diet-induced 2-3 fold increase in gut-derived plasma lipopolysaccharide (LPS) level (14), which may result in low-grade systemic and tissue inflammation, contributing to a metabolic disease phenotype (14, 15, 19–22). Metabolic endotoxemia is indicative of decreased intestinal epithelial barrier function, also referred to as “leaky gut” syndrome (**Figure 1**), which allows undesirable luminal immunogens, such as LPS, but also bacterial DNA and RNA and to some degree viable bacteria, to cross into the blood and lymphatic system (14, 29). Accordingly, studies have detected high levels of gut-derived bacterial products in the blood of obese and diabetic patients (30, 31). The intestinal barrier has since proved to be altered by additional factors including food additives (such as emulsifiers and artificial sweeteners), and contaminants (such as mycotoxins), which may in addition affect and/or change microbiota composition (32, 33).

Although many studies have reported on the role of microbiota in obesity and T2D, these mainly focused on characterization of microbiota composition and diversity in response to diet, disease and treatment (18, 23–28). Studies investigating causal relationships between microbiota and metabolic effects are lacking due to the complexity of host-microbe interactions (34–36). Emerging evidence, although scant to date, suggests that the mammalian host may shape and control their microbiota, a concept described as “ecosystem on a leash”, which is based on the evolutionary theory that host-microbe and microbe-microbe interactions play an essential role in the symbiotic relationship between hosts and their commensal microbiota (37). It has been postulated that host antimicrobial secretions, antibodies, such as immunoglobulin A (IgA), as well as gut antigen surveillance (e.g. through luminal antigen sampling by intestinal mucosal dendritic cells (DCs) or M cells), may exert this type of control on microbiota (37). Considering that the intestinal tract is exposed to an enormous antigen burden, from commensal microbiota to pathogens to food derived antigens, it is evident that the intestinal immune system is a critical role player in creating a tolerogenic milieu and preventing unwanted inflammatory responses. Thus, investigating the role of intestinal immune system and its potential in alleviating and preventing metabolic diseases through inflammatory, barrier-protective and other pathways have increasingly become focus of research, which was reviewed some years ago (38, 39).

Taken together, these studies suggest that gut health, barrier function and the intestinal immune system play an integral role in the development of metabolic disease and may place the intestines at the center and perhaps the beginning of the pathophysiology of obesity and T2D (**Figure 1**). The purpose of this review is to provide an overview of key studies that have afforded insight into how intestinal immune function, inflammation and barrier function impact on the development of obesity and T2D with specific focus on immune function and intestinal epithelial cells. Studies in animal models and humans that investigated intestinal immune cells, barrier function and/or inflammation in intestinal tissues together with effects on systemic glucose and/or insulin metabolism and/or weight gain during obesity and T2D were selected. Search terms included “intestinal barrier function”, “type 2 diabetes”, “intestinal immune system”, “obesity”, “lipopolysaccharides”, “leaky gut”, “intestinal epithelial cells”, “glucose intolerance”, “insulin resistance” and were used.

## 2 The Role of the Intestine in Metabolic Homoeostasis

Apart from its role in nutrient digestion and absorption, the gastrointestinal tract (GIT) functions as a physical barrier to prevent undesirable luminal components, such as pathogens, dietary antigens and microbiota from entering the body (40, 41). The intestinal barrier is complex and comprises multiple “layers” (see insert in **Figure 1**). A mucus layer covering the intestinal epithelium creates a physical barrier between the lumen and the tissues. Intestinal epithelial cells (IECs) secrete various antimicrobial compounds, such as lysozyme and defensins, which create a chemical barrier to prevent contact between microbiota and the tissues. The immunological barrier comprises secreted IgA, which is produced in plasma cells in the lamina propria, and a full complement of immune cells located in the lamina propria and gut-associated lymphoid tissues (42). Since the GIT is in constant contact with antigens and microbes, the gut-associated lymphoid tissues form a large part of the host immune system (43–45). Intestinal mononuclear phagocytes determine whether tolerance or an immune response is needed, depending on whether the antigens belong to commensal microbiota or pathogens (43). Antigen-presenting cells, such as tissue resident macrophages and DCs, shape the adaptive immune response (T and B cells) through secretion of cytokines and lipid mediators, which may have profound effects on the tissue microenvironment, including decreased epithelial barrier function and changing commensal microbiota composition (38, 39).

The role of the intestine in metabolic homeostasis has been mainly ascribed to enteroendocrine cells, that act as sensors for nutritional signals and produce and secrete key hormones, such as glucose-dependent insulinotropic polypeptide (GIP), glucacon-like peptide 1 (GLP-1) and peptide YY, into the circulation (46, 47). Of these hormones, GIP and GLP-1, commonly referred to as incretins, modulate postprandial glucose concentrations by inducing a rapid and strong insulinotropic effect after meal ingestion, which is blunted in patients with T2D (48) and has been widely explored as pharmaceutical target for T2D. Recently, enteroendocrine cells have been shown to sense microbial metabolites (49) and may thus serve as a link between microbiota and their metabolic effects (50). Further supporting a functional role of the intestinal tract in T2D is the phenomenon that compounds with low bioavailability, such as certain plant polyphenols or polyphenol rich plant extracts, can still elicit significant biological responses *in vivo*, such as anti-hyperglycemic effects (51–53). Accordingly, a recent review hypothesized that effects elicited in the gut might be the primary mechanism of action of polyphenols, including, for instance, modulating carbohydrate digestion and uptake, energy metabolism and interaction with microbiota (54). Similarly, a delayed-release preparation of the first-line drug for T2D, metformin was less bioavailable but as effective as “normal” metformin, suggesting an involvement of the distal bowel (ileum and colon) in its therapeutic efficacy, presumably *via* increased GLP-1 secretion from L cells and enhanced neural signaling (55).

## 3 The Intestinal Immunological Barrier in Obesity and T2D

### 3.1 Modulation of LPS and Metabolic Endotoxemia

As a membrane component of gram-negative bacteria, LPS is found in copious quantities in the gut as part of commensal microbiota and pathogens. LPS has a broad spectrum of negative health effects in the body ranging from inflammation to cancer promotion (56–58). Its lipid components are responsible for inducing the strong immune activation (59). LPS signaling, which is facilitated by pathogen associated molecular pattern (PAMP) receptors, was identified as a critical feature in intestinal homeostasis (14). The LPS-induced toll-like receptor 4 (TLR4) signaling cascade involves the co-receptor cluster of differentiation (CD) 14 as well as intracellular adaptor molecules such as Myeloid differentiation primary response gene 88 (MyD88) and TIR-domain-containing adapter-inducing interferon-β (TRIF) as key components (60–62). Metabolic endotoxemia, which refers to increased LPS levels in the blood, occurs when the intestinal barrier function is compromised (14). Several studies have reported strong associations between increased LPS levels due to intestinal barrier dysfunction and metabolic disease (63–66). In animal studies, increased LPS levels induced systemic and tissue inflammation similar to studies using high fat diet (HFD) model (14, 15). For example, when Cani et al. (14) mimicked HFD-induced endotoxemia by intraperitoneal infusion of LPS, they found significantly increased expression of inflammatory cytokines, such as interleukin (IL-) 1 in liver, adipose and muscle tissues. Furthermore, studies have associated metabolic endotoxemia with the development of non-alcoholic liver disease (67–69) as well as adipose tissue inflammation and dysfunction (58, 70), which are risk factors and/or precursors for the development of T2D. Accordingly, circulating LPS serves as a useful biomarker of compromised intestinal barrier function with augmented LPS levels often reported in obese and diabetic patients (71–73). It has been suggested that LPS plays an essential role in the onset of obesity and T2D development (14, 15). In contrast, Dalby et al. (74) showed that deletion of the TLR4 and co-receptor CD14, which are involved in LPS signaling (60, 61), did not prevent diet-induced obesity in mice, suggesting that obesity may develop independent of LPS and metabolic endotoxemia. Intriguingly, intestinal permeability and LPS levels were raised in obese TLR4 and CD14 knockout mice (74), suggesting that LPS could signal through an unidentified TLR4-independent pathway (75). Importantly, knockout of TLR4 in specific cell populations such as intestinal epithelial cells (IECs) rather than whole-body knockout may be required to induce metabolic effects (66). Lu et al. (66) recently showed that intestinal TLR4 could regulate the interaction between host and microbiota and thus affect metabolic syndrome. Using knockout of TLR4 specific to IECs or myeloid cells, these authors showed contrasting effects where deletion in IECs increased body weight and impaired glucose metabolism, whereas knockout in myeloid cells decreased body weight. Lu et al. (66) further showed that the metabolic effects of IEC-specific TLR4 knockout could be reversed by treating mice with antibiotics, suggesting that TLR4 expression in IECs play a central role in protective host-microbe interactions.

Another member of the TLR family, TLR5, plays a critical role in antigen recognition, specifically bacterial flagellin, while other roles include preserving barrier function in the gut (76). The important surveillance function of TLR5 in the intestines is supported by its location on the basolateral side of IECs, where it acts as a sensor for increased intestinal permeability (76). Vijay-Kumar et al. (77) showed that TLR5 knockout mice fed a standard chow diet developed colitis, mild inflammation and obesity. These mice also developed hypercholesterolemia, high blood pressure, hypertriglyceridemia, hyperinsulinemia and fasting hyperglycemia. TLR5 knockout mice developed T2D when these mice were fed an HFD, while food restriction prevented the increase in body weight although it could not improve glucose intolerance (77). These authors suggested that in TLR5 knockout mice, inflammatory mediators such as cytokines may inhibit the insulin signaling pathway, which may prevent a complete regain of glucose tolerance during food restriction. The important role of TLRs in obesity and metabolic disease is reiterated in that dietary fatty acids, especially saturated fatty acids supplied by HFDs, can activate TLRs in addition to LPS (78–80).

An enzyme intricately linked to endotoxemia is intestinal alkaline phosphatase (IAP), as it is involved in the detoxification of LPS (81). Knockout of IAP in mice leads to decreased intestinal barrier function and increased circulating levels of LPS and inflammatory markers (63). These mice also display glucose intolerance, insulin resistance and hyperinsulinemia. When IAP activity was diminished with a known inhibitor of IAP, such as dietary phenylalanine, impaired glucose tolerance was evident in mice fed an HFD when compared to HFD-fed control mice, while IAP knockout mice developed T2D when they were fed an HFD (63). On the other hand, IAP supplementation improved metabolic syndrome in mice and prevented LPS uptake *via* chylomicrons, leading to improved lipid profiles and liver steatosis scores (63). Malo (81) showed that fecal IAP levels are lower in diabetic compared to non-diabetic, obese patients. In contrast to Malo (81), de La Serre et al. (82) identified IAP as a key player in susceptibility to obesity. These authors showed that HFD-fed Sprague Dawley rats displayed altered microbiota composition. However, reduced IAP activity and increased endotoxemia, ileal TLR4 expression, and inflammation were observed in diet-induced obese rats compared to non-obese rats on an HFD. These findings suggest that decreased gut barrier and IAP activity are associated with obesity and that changes in the microbiota are independent of obesity, which agrees with other studies (83, 84). IAP levels decrease with age, and it was shown that supplementation of IAP in mice increased longevity and reduced frailty, supporting the notion that lower IAP activity may contribute to age-related diseases including metabolic syndrome (73).

### 3.2 Intestinal Inflammation

A healthy intestinal tract requires efficient tolerance signals to modulate and suppress inflammatory responses resulting from exposure to an overwhelming number of commensal bacteria, potential dietary antigens, and various ubiquitous enteric pathogens. This is accomplished through suppressor cells, whose tolerogenic and homeostatic functions are critical to facilitate effective barrier function and synergisms with intestinal microbiota. The intestinal immune system, therefore, requires a certain degree of isolation from the systemic immune system (85, 86). The effectiveness of this defense system is demonstrated by studies showing that early metabolic disease generates low-grade intestinal inflammation (87–89), while evidence of sub-clinical inflammatory changes, such as macrophage infiltration, has been reported (88, 90). Inflammatory signals play a crucial role in tight junction function and assembly (91), thus it is worthwhile to discuss selected key studies that specifically investigated intestinal inflammation below.

Kawano et al. (90) showed that in mice, inflammatory responses in the colon, including monocyte infiltration, loss of goblet cells as well as a decrease in crypt depth, preceded HFD-induced inflammatory changes in other tissues, such as the adipose tissue. Infiltrating colonic macrophages displayed a pro-inflammatory secretory profile after 4 weeks of HFD feeding, however, increased expression of inflammatory markers was only evident in liver and adipose tissue after 8 and 12 weeks, respectively, of HFD feeding (90). Oxidative stress was increased in the colon after 4 weeks of HFD feeding and occurred after 8 weeks in the epididymal fat depot. Intestinal inflammation preceded the development of obesity in HFD fed mice also in a study by Ding et al. (92). TNFα expression was increased in the ileum of these mice and was associated with higher body weight, fat mass and insulin resistance and 2 weeks of HFD feeding increased nuclear factor kappa B (NFκB) expression in these mice. In contrast, HFD feeding did not induce obesity and inflammation in germ-free mice, however, the inflammatory phenotype observed in conventional mice was transferrable to germ-free mice using fecal transplants (92). Taken together, these results suggest that the inflammatory effect observed in response to HFD feeding was associated with microbiota.

Changes in microbiota composition can affect LPS-induced signaling pathways including TLRs (15). In this regard, Kim et al. (19) showed that HFD-feeding in mice induced TLR4 expression, decreased intestinal barrier function and induced intestinal and systemic inflammation *via* increased pro-inflammatory cytokine production, increased cyclooxygenase-2 (COX-2) and inducible nitric oxide synthase expression. That pro-inflammatory cytokine levels were not affected by the HFD in TLR4 deficient animals (19) provided evidence for a role of gut-derived LPS signaling in HFD-induced chronic inflammation.

Using 5-aminosalicylic (5-ASA, or mesalamine), a drug with intestine-specific anti-inflammatory properties, Luck et al. (88) showed that a 12 to 14 week treatment of obese mice ameliorated HFD-induced insulin resistance through anti-inflammatory and barrier protective properties. Although treatment with 5-ASA did not inhibit weight gain, it improved liver steatosis, reduced fasting plasma glucose concentrations and improved glucose tolerance (88). The effect of 5-ASA treatment was dependent on the intestinal immune system since it showed no effect in beta7 null mice, which are devoid of intestinal immune cells. Furthermore, 5-ASA treatment improved intestinal barrier leakage, as assessed using the fluorescein isothiocyanate (FITC)-dextran assay. Overall, Luck et al. (88) demonstrated that HFD-induced inflammation in the gut and treatment with a local acting anti-inflammatory drug improved metabolic parameters, which implicated the intestinal immune system as a potential mechanism to improve metabolic function. In addition, Liu et al. (93) showed that peroxisome-proliferator-activated receptor gamma (PPARγ) played a significant role in mitigating the inflammatory effects of the HFD feeding in the colon tissue of diet-induced obese mice. These authors showed that exercise produced anti-inflammatory effects in the colon of HFD-fed mice, which a PPARγ antagonist abrogated. PPARγ expression was previously found to be reduced by HFD feeding and its reduced expression is considered an indicator of inflammation in the colon (94).

### 3.3 A Role for the Innate Immune System in Metabolic Disease

While the role of the innate immune system in adipose tissue inflammation and insulin resistance is well documented (95–99), few studies have investigated the role of the intestinal innate immune system in the development of obesity, insulin resistance and T2D. The innate immune system, which includes DCs, macrophages, innate lymphoid cells and eosinophils, is the first line of defense against pathogens, especially in the intestines, but it is also essential for shaping the adaptive immune responses (100, 101).

Innate immune cells release monocyte chemoattractant protein-1 (MCP-1), also referred to as C-C motif chemokine ligand (CCL2), which is a critical chemoattractant that initiates recruitment of immune cells to sites of inflammation. CCL2 signals through the CCL2 receptor (CCR2) which is expressed on most innate immune cells and effector cells such as T cells (102, 103). Kawano et al. (90) showed that HFD feeding induced CCL2 expression and that knockout of IEC-specific CCL2 ameliorated HFD-induced insulin resistance, infiltration of pro-inflammatory macrophages in the colon mucosa, and adipose tissue inflammation. Furthermore, macrophage-specific knockout of CCR2 in the colon improved glucose tolerance and insulin resistance in mice. In the colon, CCR2 knockout was associated with immunological changes such as the decreased production of pro-inflammatory cytokines and barrier protection through increased expression of the tight junction protein, claudin 1. These changes resulted in improved barrier function and lowered systemic LPS levels as key events in improving metabolic disturbances. The CCL2/CCR2 signaling axis may therefore promote a pro-inflammatory environment in the colon which likely increases intestinal permeability with significant adverse effects on whole-body glucose homeostasis (90).

Another component of the innate immune system, eosinophils, are traditionally thought to be hallmarks of acute inflammatory, anti-parasitic and allergic responses, such as asthma (104, 105). However, eosinophils are also found in immune cell infiltrates in inflammatory bowel diseases such as Crohn’s disease and ulcerative colitis, where eosinophil activation contributes to neutrophil recruitment and tissue ulcerations (106, 107). Findings by Johnson et al. (87) support a role for eosinophils in diet-induced metabolic disease. These authors showed that a short-term, 7-day HFD feeding regimen in mice decreased eosinophils levels in the small intestines, although no signs of inflammation, such as monocyte or macrophage infiltration, were observed. These changes were accompanied by increased permeability in the small intestine as measured by the FITC-dextran assay and fecal albumin levels, and, notably, occurred before the onset of metabolic dysfunction. Since *ob/ob* mice, which develop obesity through hyperphagia as they do not produce leptin due to a mutation, did not display similar eosinophil trafficking defects, Johnson et al. (87) concluded that this defect was caused by HFD feeding. While the significance of the HFD-induced reduction in the number of eosinophils in the intestines is not yet clear, new roles of eosinophils with focus on their involvement in intestinal tissue homeostasis are under investigation (108, 109). Emerging evidence also suggested that eosinophils may contribute to immunoglobulin A (IgA) expression (106, 110).

### 3.4 Adaptive Immune System

#### 3.4.1 B Cells

IgA is the most abundant antibody subtype produced by B cells in the intestines and is generally considered the first line of defense (111). IgA is mainly secreted across mucosal surfaces, such as in the intestinal mucosa, where it fulfils several essential functions including maintaining gut homeostasis and microbial tolerance. Secretory IgA can bind to or “coat” microbiota, thereby inducing immune exclusion and neutralization by altering bacterial motility and gene expression, and facilitating antigen uptake (112–116). A recent study reported that fecal IgA levels did not differ between non-obese patients with T2D and healthy controls or patients with Crohn’s disease (117). However, these authors found a significant decrease in IgA expressing B cells in the intestinal mucosa of non-obese patients with T2D, specifically in mucosal CD19^+^CD20^-^ cells, compared to healthy patients. These changes were accompanied by an increase in IgG expressing B cells, which may be linked to increased T cell-derived interferon-gamma (IFNγ) secretion. These authors suggest that plasma cells of non-obese T2D patients are more reactive to IFNγ compared to healthy controls, resulting in a pro-inflammatory environment and depletion of IgA^+^ cells in intestinal tissues (117).

In mice, IgA^+^ plasma cells were decreased in the large intestines of HFD-fed obese mice compared to normal-weight mice fed a standard diet, which was accompanied by increased insulin resistance and higher fasting plasma glucose concentrations (118). These results suggest a link between the abundance of IgA^+^ plasma cells and glucose metabolism in response to HFD feeding. Furthermore, lower intestinal IgA secretion was associated with altered microbiota composition and produced a microbiota signature eliciting a transplantable metabolic phenotype when transferred into germfree or antibiotic-treated mice. It was proposed that lower IgA levels and increased bacterial IgA binding affinity in response to HFD feeding stimulate or induce pathogenic bacteria to migrate across the intestinal barrier. Interestingly, metformin treatment or bariatric surgery improved IgA deficiency in this model (118). In a recent study by Sakamoto et al. (119), HFD feeding decreased IgA^+^ cells in the lamina propria of the small intestines, which was associated with increased body weight and fasting blood glucose concentrations compared to mice fed a standard chow diet. IgA, particularly in intestinal tissues, has not yet been the focus of in-depth studies investigating the pathophysiology of T2D. However, the highlighted effects on regulating glucose homeostasis and microbiota composition suggest that it could be an important target for prevention of metabolic diseases such as obesity and T2D. Further studies investigating its role in the pathophysiology of metabolic diseases are therefore warranted.

#### 3.4.2 T Cells

The role of different T cell subsets in intestinal immune homeostasis and metabolic diseases is the subject of intense research (85, 114, 120). T-helper (Th)17 cells, intraepithelial T lymphocytes, regulatory T (Treg) cells and the cytokines and cytokine receptors involved in their differentiation have emerged as targets of interest in metabolic disease. T cells set the inflammatory tone, with each subset of T-helper cells secreting distinct cytokine profiles. Monteiro-Sepulveda et al. (121) attempted to eliminate diet as a confounding factor by studying intestinal inflammation in metabolically healthy obese as well as obese patients with T2D. These authors showed that obesity-induced inflammation in the jejunum resulted in increased CD3^+^ T cells in the jejunal epithelium. The number of macrophages, CD3^+^ T cells and intraepithelial T cells was increased in the jejunum of obese patients, while mature DCs and natural killer cells were increased in obese patients with T2D (121). Increased epithelial T cell and innate immune cell populations were linked to intestinal and specifically jejunal inflammation in obesity and correlated with high dietary fat content. In addition, the IECs displayed insulin resistance due to T cell activation (121). The link between increased pro-inflammatory immune cell populations and reduced insulin sensitivity in IECs in obesity could provide new insight into the development of insulin resistance and T2D. Other studies have reported that diet-induced obesity is characterized by the increased presence of pro-inflammatory intestinal Th1 and CD8^+^ cells and reduced Treg cells (88). In the latter study, 3 weeks of HFD feeding changed the immune cell populations in the lamina propria of the colon towards a pro-inflammatory milieu, resulting in decreased Treg cells and increased IL-17 producing γδ T cells (88). After 14 weeks of HFD feeding these effects were also observed in the small intestine, with an increase in Th1 cells producing IFNγ and a decrease in CD4^+^/Forkhead box protein P3 (FoxP3)^+^ Treg cells. Reduced Treg cells and increased T-box protein expressing T cells (T-bet^+^ cells) and CD8^+^ cells were observed in the colon and small intestines of obese patients, while no histological signs of inflammation were apparent (88). Interestingly, Luck et al. (88) showed that beta7 null mice, which are devoid of intestinal immune cells, displayed improved glucose metabolism in response to a 12 week HFD-feeding regimen when compared to wild-type mice, without beneficial effects on body weight gain. The authors suggested that induction of disturbances in glucose metabolism and insulin resistance may therefore be dependent on gut immune system dysfunction (88). While Luck et al. (88) assessed T cell populations in the lamina propria of the small and large intestine, a recent study documented a critical role of intestinal intraepithelial αβ and γδ T cells in whole-body glucose and lipid metabolism (122). These authors showed that mice lacking integrin β7 were protected against diet-induced obesity and atherosclerosis with improved glucose tolerance and displayed reduced numbers of intraepithelial T cells. As these T cell subsets represent the major cell population expressing GLP-1 receptors, He et al. (122) were able to explain these positive metabolic effects in mice lacking integrin β7 through increased bioavailability of GLP-1 leading to increased glucose disposal.

While Th1 and Th2 cells are involved in shaping active immune responses, the main function of Treg cells is to create (self)tolerance, which is particularly important in the gut due to the high antigen load (123). Everard et al. (124) showed that FoxP3 expression was increased in the jejunum of MyD88 knockout mice fed an HFD compared to HFD-fed wild type mice, likely due to the key role of MyD88 in the TLR and LPS signaling pathway. MyD88 is an intracellular adapter protein that facilitates TLR and IL-1 receptor signaling and is, therefore, a critical link in the activation of pro-inflammatory transcription factors such as NFκB or mitogen-activated protein kinases (125). Compared to wild type mice, FoxP3 expression in Treg cells was increased in the colon of MyD88 knockout mice fed either a standard diet or HFD. Everard et al. (124) further showed that knockout of MyD88 in selected cell types such as in myeloid cells conferred no protection against obesity, while IEC-specific knockout of MyD88 played a pivotal role in development of T2D and obesity, likely through sensing diet-related stress (124). Epithelial MyD88 knockout also affected microbiota composition and transferring these microbiota to germfree mice conferred the positive metabolic traits of the MyD88 knockout, suggesting that microbiota can be shaped by the host (124).

Th17 cells, initially thought to induce pro-inflammatory responses, are now recognized to exert contrasting protective and pathological roles (126). Th17 cells may protect against certain infections (126) and in the small intestine, they have been implicated in maintaining microbiota homeostasis. A study by Hong et al. (127) showed that *ob/ob* mice and HFD fed mice displayed lower abundance of Th17 cells in their small intestines, which was accompanied by increased T-helper 1 (Th1) cell-derived IFNγ levels compared to Th17 cell-derived IL-17 and IL-22. However, increased abundance of Th17 cells improved metabolic status (127). Garidou et al. (128) showed that HFD feeding in mice influenced intestinal T cell populations. Feeding a diet consisting of 72% fat and devoid of carbohydrates for 10 and 30 days did not induce weight gain, but these animals developed glucose intolerance and insulin resistance with a concomitant loss of Th17 cells in the ileum, which preceded inflammation in adipose tissues. In the ileum and mesenteric lymph nodes, IL-17, IL-22 and IL-10 gene expression was reduced, while a loss of Th17 cells (i.e. IL-17 and IL-22 secreting) was detected in the colon and small intestinal tissues (128). Treg cell numbers were lower in the small intestinal lamina propria in HFD fed mice, with a proportional increase in Th1 cells. In a study using nucleotide oligomerization domain (NOD)2 knockout and wild type mice, Th17 responses were decreased in response to HFD feeding (129). Wild-type and NOD2 knockout mice displayed reduced neutrophil-dependent oxidative bursts against bacteria following HFD feeding, which could play a role in shaping microbiota composition (129). It seems that metabolic disease could be characterized by reduced Th17 responses in the intestines, in contrast to inflammatory bowel disease where increased IL-17 was associated with increased inflammation, disease severity and relapse (130, 131).

##### 3.4.2.1 Role of Th17 Specific Cytokines

Th17 cells have been identified as a distinct cell lineage due to their cytokine secretion profiles, including IL-17, IL-22 and IL-23 (132), and were shown to fulfil critical functions in the defense against pathogens in the gut mucosa (133). The shaping of gut microbial populations is accomplished by producing antimicrobial peptides, which can be stimulated by Th17 cytokines such as IL-22 (134, 135). IL-22 is part of the IL-10 family and is expressed by Th17 cells, innate lymphoid cells and Th22 cells. Its protective and regenerative functions have been demonstrated in various tissues, including the gut (136, 137). IL-22 deficient mice develop metabolic syndrome when fed an HFD and treatment with an IL-22Fc fusion protein ameliorated body weight gain and glucose intolerance. However, continued treatment was required to maintain the therapeutic effect (138). Treatment with the IL-22Fc fusion protein also induced changes in microbiota composition, however, since this did not occur in mice fed the control diet, the effect of the IL-22Fc on microbiota was suggested to be HFD-dependent and secondary to metabolic changes (138).

To investigate intestinal inflammation, Gulhane et al. (65) used Winnie mice, who contain a mutation in the *Muc2* gene, which results in misfolding of the mucin 2 glycoprotein, which is the main component of the intestinal mucus layer. Winnie mice are considered a model of endoplasmic reticulum stress-induced colitis, which constitutes an epithelial defect, while mice present with a normal immune system. HFD feeding aggravates colitis in Winnie mice, but when these mice were treated with IL-22, the intestinal mucosa produced lower cytokine levels in response to HFD, which increased claudin 1 protein expression, thereby improving tight junction function, and decreasing systemic LPS levels. IL-22 treatment further normalized the changes in the microbiota induced by HFD, for instance by increasing levels of *Akkermansia muciniphila* and decreasing levels of *E. coli* (65).

IL-23 is a member of the IL-12 family, which is associated with chronic inflammatory disease and is considered an important inducer of Th17 cells (139, 140). Interestingly, IL-23p19 knockout mice gained more weight compared to wild type mice in response to HFD feeding, as shown by total fat accumulation and a trend towards increased visceral adipose tissue (141). Furthermore, HFD feeding increased insulin resistance, glucose intolerance and fasting plasma glucose levels in IL-23 deficient animals compared to the wild type animals on HFD (141). These results suggest that IL-23p19 could have protective properties during diet-induced obesity.

## 4 Impact of Spatial Segregation in the Gut on Obesity and T2D

The intestinal barrier (compare insert in **Figure 1**) is a complex system comprising (i) an immunological barrier, which consists of immune cells, cytokines and IgA as discussed in the previous section, (ii) a chemical barrier, which is characterized by antimicrobial secretions and (iii) a physical barrier, which involves spatial segregation through mucus and epithelial cells that provide a barrier through intercellular tight junctions. The intestinal barrier and its permeability play an important role in the pathogenesis of obesity and T2D, however, it is still unclear whether increased intestinal permeability is a cause or consequence of metabolic disease, or perhaps both (34–36). Nevertheless, the critical role of the intestinal barrier in metabolic disease development and prevention is becoming increasingly recognized and mechanisms that prevent pathogens, microbiota and microbiota-derived products from gaining access to the circulation through breaching intestinal tissues will be discussed in the following section.

### 4.1 Chemical Barrier

Antimicrobial molecules produced by the intestinal epithelium are important defense mechanisms to prevent infection and dysbiosis of commensal microbiota. HFD feeding was shown to decrease the expression of antimicrobial peptides (124, 128, 142–145). The gene expression of the antimicrobial peptide regenerating islet-derived protein 3 gamma (RegIIIγ) was reduced in the jejunum and colon of HFD-fed wild-type mice, but not in HFD-fed MyD88 knockout mice (124). This is of interest as RegIIIγ has been suggested to modulate microbiota through spatial segregation, thereby preventing commensal microbiota from reaching the epithelium (124, 146, 147).

In contrast to studies that reported decreased antimicrobial peptides in response to HFD feeding (124, 128, 142–145), Gao et al. (148) showed that HFD feeding increased the expression of antimicrobial peptides in the colon, which was counteracted by their treatment with polyphenols derived from Pu-erh tea. This inconsistency between these studies regarding HFD-induced antimicrobial peptide expression may be due to diet composition. Variation in diet composition between studies poses a major challenge in microbiota research as differences in diet composition, particularly regarding fat and fiber content, can induce variable metabolic outcomes (149, 150). While both Everard et al. (124) and Gao et al. (148) provide information on fat content (60% and 45% of kcal as fat, respectively), the type of fat and fiber in the diets were not reported. It is tempting to speculate that the type of fat may affect intestinal barrier function and, although there is limited evidence, diets rich in anti-inflammatory omega 3 fatty acids are likely to display barrier protective effects and/or modulate microbiota composition (151–153). Dietary fiber is an important source for fermentation by selected microbiota. High fiber diets may, therefore, profoundly affect microbiota composition through selecting strains that produce beneficial microbial metabolites such as short chain fatty acids (SCFA), which can be used as energy sources by enterocytes. If a standard diet containing grain-based fiber is compared to an HFD lacking fiber, metabolic benefits cannot be directly compared (149, 150).

### 4.2 Physical Barrier

In addition to their critical function in nutrient absorption, IECs also form a physical barrier designed to segregate the internal system from the external luminal environment that is rich in antigens and microbes. The intestinal epithelium consists of a single layer of cells where the intercellular spaces are locked with tight junction complexes, which can be used to regulate permeability towards small solutes such as water, electrolytes and macromolecules (154). Tight junction complexes play a critical role in barrier function and modulation and have been extensively reviewed recently (155–157). HFD feeding alters tight junction function and increases intestinal permeability. Gulhane et al. (65) showed that the tight junction protein claudin 1 was significantly decreased after 11 weeks of HFD feeding, which was accompanied by increased mucosal permeability and circulating levels of LPS. Suzuki and Hara (158) showed that HFD feeding increased intestinal permeability in lean and obese rats, which was linked to decreased expression of the tight junction proteins claudin 1 and 3 as well as junctional adhesion molecule 1 (JAM-1). These authors used the obese Otsuka Long Evans Tokushima Fatty (OLETF) rat model, in which rats become hyperphagic due to knockout of the cholecystokinin receptor, which senses cholecystokinin that is released from I cells in the small intestine and regulates food intake and satiety through neuronal feedback to the brain (159). HFD feeding induced intestinal inflammation in OLETF rats, however, the role of endotoxemia as a driver of this inflammatory response was questioned as, although systemic LPS levels were increased, systemic cytokine levels remained unchanged (158).

Goblet cells are the main source of the mucus that forms a physical barrier, shielding the intestinal epithelium from luminal antigens and microbiota (160, 161). Everard et al. (162) reported that HFD feeding in mice decreases the intestinal mucus layer, which improved after supplementation with *Akkermansia muciniphila*, an interesting and well-researched beneficial member of the microbiota (163). Another study showed that dietary prebiotics, which provide nutrients for selected microbiota and therefore support growth of selected commensals, are able to increase the number of goblet cells and thus mucus layer thickness (164), which is an integral part of the healthy intestinal barrier (165). Goblet cell and barrier dysfunction as well as inflammation following HFD feeding was recently linked to non-esterified fatty acid (NEFA) levels, and specifically to palmitate-induced oxidative and endoplasmic reticulum (ER) stress in goblet cells (65). This resulted in the thinning of the mucus layer and increased expression of non-O-glycosylated mucin 2 precursors in the intestines.

#### 4.2.1 Intestinal Epithelial Cells (IECs) and Their Functional Diversity

**Figure 2:**
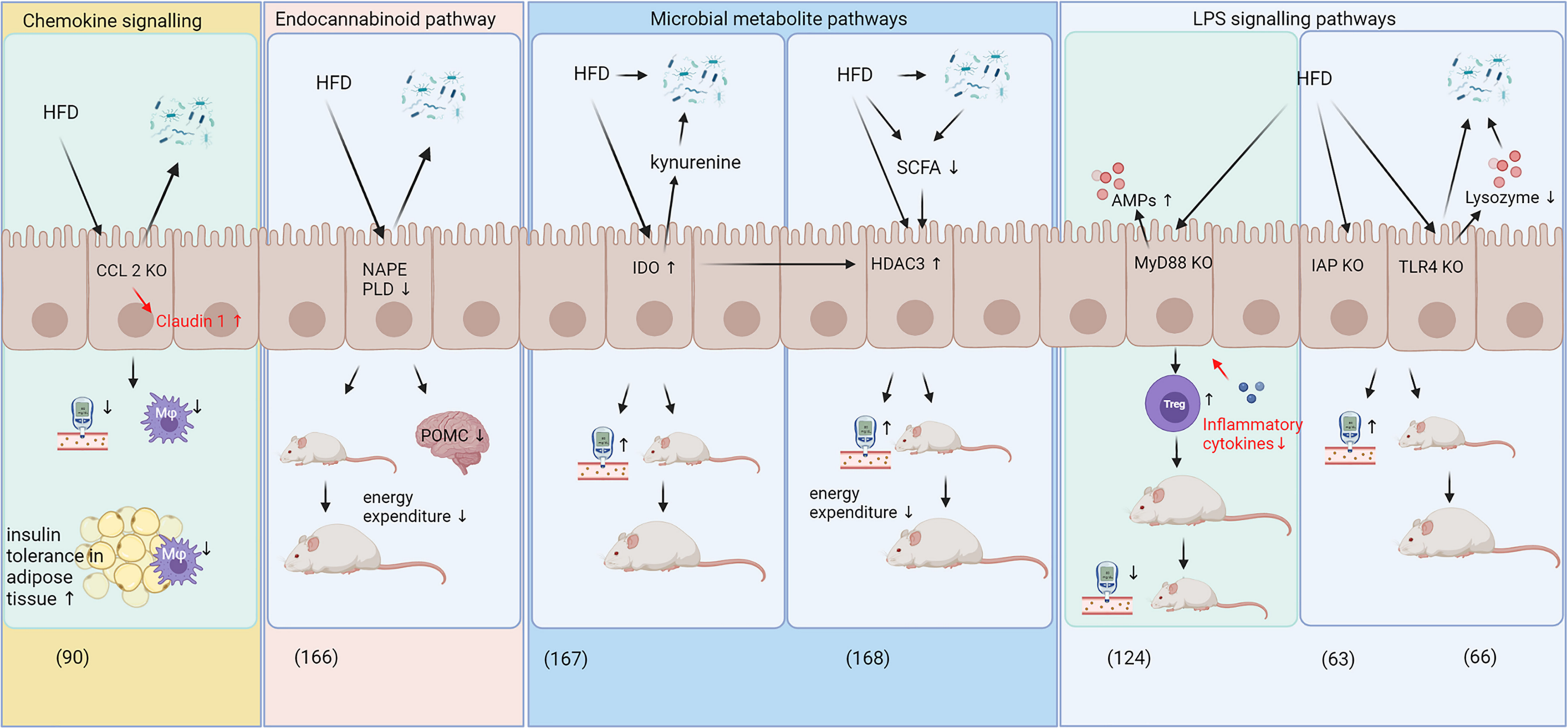
Specific and diverse roles of IECs in HFD-induced metabolic disease. The roles of intestinal epithelial cells (IECs) in body weight gain and glucose homeostasis can be categorized into immune crosstalk/chemokine signaling (90), pathways involving the endocannabinoid system (166), pathways involving microbial metabolites (167, 168) and signaling pathways related to LPS signaling (63, 66, 124). While most pathways exerted adverse effects on glucose metabolism and weight gain in response to HFD feeding, IEC specific knockout of CCL2 and MyD88 decreased body weight gain and improved glucose metabolism during high-fat diet feeding (90, 124). CCL2 and MyD88 knockout improved intestinal barrier function by increasing claudin 1 levels and decreased expression of inflammatory cytokines, respectively (highlighted in red). AMPs, antimicrobial peptides; CCL2, C-C motif chemokine ligand 2; HDAC3, histone deacetylase 3; HFD, high fat diet; IAP, intestinal alkaline phosphatase; IDO, Indolamine 2;3-dioxygenase; IEC, intestinal epithelial cells; KO, knockout; LPS, lipopolysaccharides; Mφ, macrophages; MyD88, myeloid differentiation primary response gene 88; NAPE PLD, N-acylphosphatidylethanolamine phospholipase D; POMC, pro-opiomelanocortin; SCFA, short chain fatty acids; TLR4, toll-like receptor 4; Treg, regulatory T cells.

IECs play a central role in intestinal barrier function as they provide a single cell layer barrier with intercellular spaces tightly locked by tight junctions, as previously highlighted. IECs produce a variety of effector molecules, e.g. they are the main cell type to express IAP, which plays a critical role in ameliorating metabolic disease through modulating LPS levels (63). Everard et al. (124) characterized a role for IEC-specific MyD88 expression in glucose homeostasis as MyD88 is an important part of the LPS-signaling cascade resulting in inflammation, while Lu et al. (66) used a TLR4 knockout mouse model to show the critical role of this receptor in metabolic health. Kawano et al. (90) demonstrated the role of the CCL2/CCR2 signaling axis in IECs and showed the importance of immune crosstalk between IECs and underlying immune cell populations in whole-body glucose homeostasis (**Figure 2**). Additional functions of IECs explaining their pivotal role in metabolic disease are discussed in this section and summarized in **Figure 2**.

Recent work has linked IECs with epigenetic mechanisms. Whitt et al. (168) showed that mice with histone deacetylase 3 (HDAC3) knockout in IECs were resistant to diet-induced obesity and showed improved glucose and lipid metabolism compared to wild-type mice, which may be due to increased energy expenditure, heat production and oxygen consumption (168). The study further showed that modulation of HDAC3 expression in IECs affected metabolically active tissues, such as the liver and adipose tissue and that an HFD reduces SCFAs (168), which are known to inhibit HDAC3 activity a (169, 170). The HFD feeding was suggested to induce HDAC3 activity, which was associated with increased body weight gain and blood glucose concentrations (**Figure 2**), while supplementation with butyrate, a SCFA, was able to reduce weight gain in HFD fed mice, presumably *via* HDAC3 dependent mechanisms. Taken together, these results suggest that HDAC3 may be implicated the development of obesity and T2D (168).

In addition to inhibiting HDAC3, microbiota-derived butyrate can also modulate indolamine 2,3-dioxygenase (IDO) activity in IECs (171). As a key enzyme in tryptophan catabolism, IDO converts tryptophane into kynurenine and is recognized as a modulator of immune function (172). IDO is expressed in macrophages, DCs and IECs and it contributes towards an immune suppressive and anti-inflammatory milieu (172) although a more complex role in intestinal homeostasis has been proposed recently, including adverse effects in metabolic disease (171, 173). Laurans et al. (167) demonstrated that IDO knockout mice were protected against HFD-induced body weight gain and displayed improved glucose tolerance. While Laurans et al. (167) did not use IEC-specific knockout, they demonstrated that IDO knockout in non-myeloid cells provides this metabolic benefit. These non-myeloid cells include IECs as the main cell type expressing IDO in the gut (171). In addition, Laurans et al. (167) showed that HFD feeding in wild-type mice increased IDO activity in the small and large intestines and this increase in kynurenine in the intestines may deplete indole derivatives that would otherwise be available for metabolism by the gut microbiota and may consequently exert a selective pressure on microbiota (**Figure 2**). The microbiota-derived indole-3-acetic acid is also an activator of the aryl hydrocarbon receptor (AHR), which, in addition, contributes to the protection against diet-induced obesity and intestinal barrier permeability (174). AHR plays a role in Treg cell differentiation (175) and a wide range of bioactive plant compounds, such as polyphenols, can act as AHR agonists and antagonists (176). Several reports have highlighted the role of the intestinal AHR, which is mainly expressed in IECs, in diet-induced obesity and metabolic disease (177–179).

IECs have been also investigated with regards to their role in the endocannabinoid system. Everard et al. (166) showed that knockout of N-acylphosphatidylethanolamine phospholipase D, a critical enzyme in endocannabinoid production, in IECs decreased energy expenditure and increased fat accumulation in response to HFD feeding without altering inflammatory status in adipose tissues or glucose metabolism. These authors showed that supplementation with *A. muciniphila* reversed most of the HFD-induced effects on N-acylphosphatidylethanolamine phospholipase D knockout mice. The endocannabinoid system plays a critical role in whole body energy metabolism (180), which was reviewed comprehensively by Veilleux et al. (181) and others (182, 183). Thus, these results suggest a significant role of endocannabinoids in intestinal tissues and in whole-body energy and glucose metabolism in relation to obesity and T2D development.

## 5 Intestinal Barrier and Immune System Play an Integral Part in the Pathophysiology of Obesity and T2D

**Figure 3:**
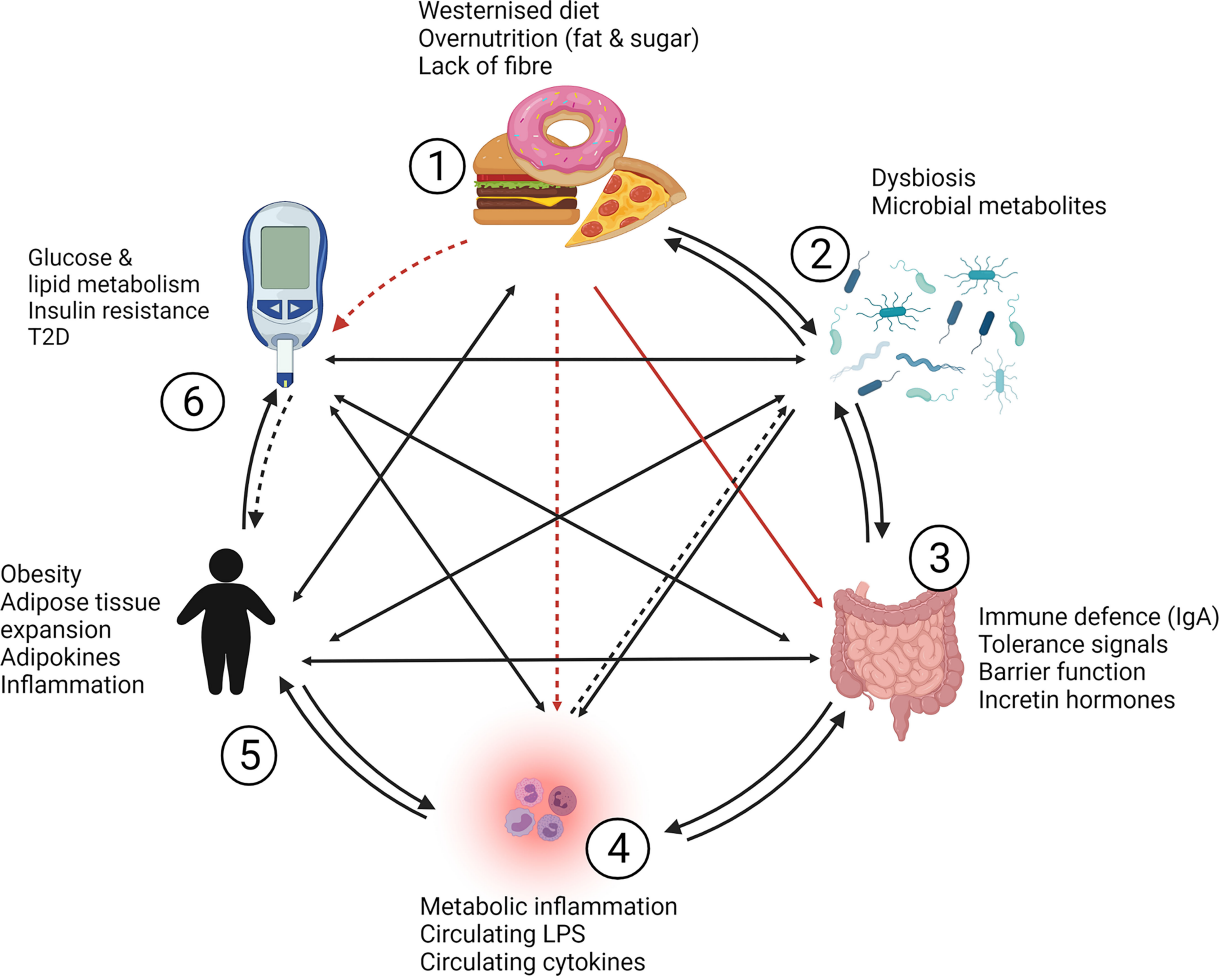
Complexity and cross-talk between contributors to obesity and T2D. Westernized diets [1], gut microbiota [2], the intestinal barrier and immune system [3] and metabolic inflammation [4] play a significant role in the development of obesity [5] and type 2 diabetes [6]. **[1]** Diet likely exerts a “one way effect” (indicated by red arrows) on glucose homeostasis, while high fat diets also affect the intestinal immune system and barrier function which can lead to [4] metabolic inflammation (indirect effects are indicated with dashed arrows). **[2]** Changes in microbiota composition and diversity have been associated with obesity, dysregulated glucose metabolism and T2D, with metabolic inflammation, and the intestinal immune and barrier system. Conversely, microbiota may also affect the dietary intake through inducing behavioral changes and cravings. **[3]** The intestines are known to shape microbiota and facilitate metabolic inflammation through barrier defects, but the intestines can also directly affect body weight and glucose homeostasis through gut hormone production and *via* expression of effector molecules and pathways in intestinal epithelial cells. **[4]** Metabolic inflammation contributes to obesity and T2D development through inducing insulin resistance. How metabolic inflammation may directly affect microbiota is not yet clear, but may occur indirectly through effects on intestinal barrier and immune function. **[5]** Obesity contributes to metabolic inflammation, but is associated with intestinal inflammation, barrier dysfunction and altered microbiota. Obesity may also influence diet and/or food intake through dysregulation in signaling related to satiety. A potentially direct effect of obesity on T2D development may involve the increased release of free fatty acid from adipose tissue which can induce insulin resistance through oxidative stress and lipid metabolites. **[6]** Type 2 diabetes and dysregulated glucose homeostasis may induce obesity in the sense that insulin resistance and hyperglycemia lead to increased lipogenesis and lipid storage but a direct pathway to increased body weight is likely the result of anti-diabetic drugs and their side effects. Hyperglycemia may induce metabolic inflammation directly *via* increased oxidative stress and indirectly through decreasing intestinal barrier function and altered microbiota composition. IgA, immunoglobulin A; LPS, lipopolysaccharide; T2D, type 2 diabetes.

Metabolic diseases, such as T2D, are complex diseases. Decades of dedicated research on T2D have advanced our understanding of the pathophysiology and keep producing advances in treatment options that improve lives. However, the complexity of disease mechanisms and the lack of a definitive cure for T2D suggest that our understanding of the disease is not yet complete. **Figure 3** outlines the crosstalk between risk factors and key players in T2D development, which include diet, microbiota, intestines, metabolic inflammation and obesity. Two-way and indirect interactions between these key players add to the complexity of the disease:

1. Nutrient dense, high fat and high sugar diets likely exert a “one way effect” on glucose homeostasis as it is well established that chronic consumption of a western diet can lead to T2D (184). High fat diets also affect the intestinal immune system and barrier function, which can lead to metabolic inflammation (14, 15).
2. Changes in microbiota composition and diversity have been associated with obesity, dysregulated glucose metabolism and T2D, with metabolic inflammation (15, 23) and modify the intestinal immune and barrier system (152, 185). Conversely, microbiota may also affect the dietary intake through inducing behavioral changes and cravings (186, 187).
3. The intestines are known to shape microbiota through the intestinal immune system, antimicrobial and antibody secretions, which are critical components of the barrier function (37, 124, 129, 152). Barrier defects and dysfunction of the intestinal tissue can facilitate metabolic inflammation (14), but the intestines can also directly affect body weight and glucose homeostasis through the secretion of gut hormones (46, 47) and *via* activation of various inflammatory and pattern receptor signaling pathways in intestinal epithelial cells (63, 66, 82, 90, 142, 166–168).
4. Metabolic inflammation was shown to contribute to obesity (14, 18) and T2D development through inducing insulin resistance, which has been reviewed extensively (8–11). How metabolic inflammation may directly affect microbiota is not yet clear, however, systemic inflammation has negative effects on barrier function through the action of pro-inflammatory cytokines (154) and may therefore indirectly influence microbiota composition or diversity through its effect on the intestinal barrier and immune system.
5. That obesity contributes to metabolic inflammation is well established (95, 97, 99, 188), but obesity has also been associated with intestinal inflammation and barrier dysfunction (121, 189) and altered microbiota (27, 190). Obesity may influence diet and/or food intake through dysregulation in leptin and satiety levels (191). A more direct effect of obesity on T2D development occurs likely through increased release of free fatty acid from adipose tissue which can induce insulin resistance through oxidative stress and lipid metabolites (192).
6. T2D and dysregulated glucose homeostasis may affect obesity in the sense that insulin resistance and hyperglycemia lead to increased lipogenesis and lipid storage (192) although increased body weight may predominantly result from side effects of antidiabetic drugs. Hyperglycemia may induce metabolic inflammation directly *via* mechanisms related to increased oxidative stress (193) and indirectly through decreasing intestinal barrier function (194). Altered microbiota composition has been documented in type 2 diabetic patients (27).

Overall, this complex interactive system between organ structures and risk factors could provide an explanation why causal relationships between changes in microbiota composition, intestinal tissue and barrier effects and metabolic health have been difficult to attain.

## 6 Summary and Conclusion

There is a paucity of knowledge about the role of intestinal immunity and intestinal barrier function in the development of metabolic disease. This review highlights that the intestinal immune and barrier system can be a powerful modulator of diet-induced inflammation, glucose homeostasis, obesity and T2D development. Given the importance of the intestinal system as the “first responder” for dietary nutrients, antigens, commensals and toxins, future research on intestinal tissue and immune homeostasis should address changes in the intestinal immune system, including Treg cells, Th17 cells, as well as IgA producing plasma cells and their secretory profiles. Studies on innate and adaptive immune cell populations have provided compelling evidence for the contribution of these cell populations in the pathogenesis of metabolic disease (87, 88, 90, 117, 118, 127, 128), however, many questions still remain unanswered.

This review also highlighted the complexity of interconnected risk factors, organ systems and their cross-talk in metabolic disease, summarized in **Figure 3**. Host immune cells can shape commensal microbiota (37, 124) and inflammatory tone. The subtle inflammatory changes in the intestines in the early stages of metabolic disease development (87–89) suggest that inflammation in the intestinal sense could be viewed as shift in immune cell populations whose cytokine profiles set either an inflammatory or tolerant “tone”, e.g. by recruiting regulatory T cells (88, 90, 127). IgA represents the initial defense that the body offers against invading pathogens and dysbiosis (195) and studies have associated IgA producing cells with microbiota composition and metabolic effects (117, 118), future research should characterize the role of IgA in obesity metabolic disease in more detail.

One way to improve intestinal barrier function may be through focusing on IECs, as they possess more diverse and vital functions in addition to nutrient and electrolyte transport than previously known. In **Figure 2**, some of the recently discovered underlying mechanisms of how IECs contribute to metabolic disease have been highlighted. Exactly how this crosstalk between IECs, the intestinal immune system and metabolically active tissues is facilitated and translates into improved metabolic phenotype is not yet fully elucidated, but the intestines may play a pivotal role in the development of metabolic diseases and deserve further investigation. This may indeed place the intestines, including the intestinal immune system and IECs, at the center of metabolic disease development and provide a new focus area to develop improved treatments for obesity and T2D in future.

## Author Contributions

Conceptualization, SR, CP, and RJ. Writing—original draft preparation, SR. Writing—review and editing, SR, CP, RJ, JL, and CM. All authors have read and agreed to the published version of the manuscript.

## Funding

SR received funding from the South African Medical Research Council (baseline funding), the South African Rooibos Council and the National Research Foundation (NRF) of South Africa, grant UID 121919. CM received funding from the NRF of South Africa under the IRG-Taiwan/South African Research Cooperation Programme, grant UID 121232.

## Conflict of Interest

The authors declare that the research was conducted in the absence of any commercial or financial relationships that could be construed as a potential conflict of interest.

## Publisher’s Note

All claims expressed in this article are solely those of the authors and do not necessarily represent those of their affiliated organizations, or those of the publisher, the editors and the reviewers. Any product that may be evaluated in this article, or claim that may be made by its manufacturer, is not guaranteed or endorsed by the publisher.
